# Adrenal cortex tumors: clinical features and laboratory finding

**DOI:** 10.1186/1687-9856-2013-S1-P110

**Published:** 2013-10-03

**Authors:** Can Thi Bich Ngoc, Vu Chi Dung, Bui Phuong Thao, Nguyen Ngoc Khanh, Tran Ngoc Son, Nguyen Thanh Liem, Nguyen Phu Dat, Nguyen Thi Hoan

**Affiliations:** 1 National Hospital of Pediatrics, Hanoi, Vietnam; 2 Hanoi Medical University, Hanoi, Vietnam

## 

The adrenal cortex tumors (ACT) include both malignant adrenal cortex cancers and benign masses that can be either secreting, of one of the hormones normally produced in the adrenal cortex or non-secretory. We describe clinical features and laboratory finding of patients with adrenal cortex tumors. This is a case series report. 29 cases of childhood with adrenal cortex tumors treated at the Vietnam National Hospital of Pediatrics in a period of 1995–2011 have been reviewed in detail the presenting features and laboratory. The study recruited 15 boys and 14 girls. The median age at diagnosis was 4.97 ± 3.61 yrs (range, 20 days to 14 yrs); 19 patients were younger than 5 yrs. Hypertension and cushingoid features were common in patients (62%, 58.6%), virilization was presenting feature in 44.8% of the group. An abdominal mass was palpable in 20.7 % of the patients. High level of testosterone was common in patients (94.1%). 30% of the patients had hypercotisonemia. All tumors were unilateral; the right adrenal glands predominated over left (1.9 :1). 62.5 % tumors over 4 cm. 22 patients had received operation to remove the tumors: 11 carcinoma, 2 adenocarcinoma. In conclusion, high percentage of patients had malignant tumors among ACT operated patients.

